# Local Urticarial Reaction Above the Site of an Intravenous Cannula: Possible Allergy to Remimazolam in a Fourteen-Year-Old Adolescent

**DOI:** 10.14740/jmc5296

**Published:** 2026-03-27

**Authors:** Caleb Blake, Marco Corridore, Sarah Khan, Brittany L. Willer, Joseph D. Tobias

**Affiliations:** a Ohio University Heritage College of Osteopathic Medicine, Athens, OH, USA; b Department of Anesthesiology and Pain Medicine, Nationwide Children’s Hospital, Columbus, OH 43205, USA; c Department of Anesthesiology, The Ohio State University College of Medicine, Columbus, OH, USA

**Keywords:** Remimazolam, Allergy, Allergic reactions, Anaphylaxis

## Abstract

Remimazolam is an intravenous, ultra-short acting benzodiazepine that has been most commonly used for procedural sedation. It received approval for procedural sedation in adults by the United States Food and Drug Administration (FDA) in 2020. Despite an overall favorable safety profile, anecdotal experience has suggested a potential for allergic phenomena, including anaphylaxis. We present a 14-year-old patient who developed a localized reaction with urticaria in an extremity following the administration of remimazolam. Although we cannot definitively identify remimazolam as the cause of the hypersensitivity reaction and no formal allergy testing was completed, the temporal association of its administration with the development of the local reaction suggests a possible relationship. Previous reports of allergic reactions following the administration of remimazolam are reviewed, mechanisms discussed, and guidelines for clinical care presented.

## Introduction

Remimazolam is an intravenous, ultra-short acting benzodiazepine that has been used most commonly during procedural sedation [1]. Following approval and introduction for clinical use during upper gastrointestinal endoscopy in China in 2019, it subsequently received approval for procedural sedation in adults by the United States Food and Drug Administration (FDA) in 2020. Although its sedative properties parallel those of midazolam, structurally remimazolam contains an ester linkage that allows hydrolysis by nonspecific tissue esterases into inactive metabolites, resulting in rapid onset and elimination with a limited context-sensitive half-life. This yields a duration of action that is short and predictable, making it easily titratable by continuous infusion [2].

Clinical experience has demonstrated that remimazolam is safe and effective, with a limited adverse effect profile. A comprehensive review by Zhang et al of adult studies comparing clinical outcomes with propofol, demonstrated that remimazolam had a decreased incidence of injection pain, respiratory depression, post-induction hypotension, bradycardia, and postoperative nausea and vomiting [3]. Despite the overall favorable profile of remimazolam, anecdotal reports have emerged suggesting a potential for allergic phenomena including anaphylaxis. We present a 14-year-old adolescent who developed localized rash and urticaria in an extremity following the intravenous administration of remimazolam. Previous reports of allergic reactions following the administration of remimazolam are reviewed, mechanisms discussed, and recommendations for clinical care presented.

## Case Report

Review of this case and presentation in this format followed the guidelines of the Institutional Review Board of Nationwide Children’s Hospital (Columbus, Ohio).

The patient was a 14-year-old (weight 55.2 kg, height 163 cm) adolescent male presenting for cardiac catheterization with coronary angiography and biopsy. His past medical history included tetralogy of Fallot repair with multiple surgical interventions, ultimately requiring orthotopic heart transplantation. His immunomodulating regimen included tacrolimus, sirolimus, and intermittent doses of subcutaneous immune globulin. Other regularly administered home medications included albuterol sulfate, atorvastatin, enalapril maleate, fluticasone propionate, azithromycin, and aspirin. Following the application of standard monitors and placement of a peripheral intravenous cannula (following skin preparation with topical chlorhexidine), the patient was premedicated with intravenous midazolam (2 mg). This was followed by an intravenous bolus of dexmedetomidine (8 µg) and an intravenous remimazolam infusion, starting at 10 µg/kg/min. Four minutes later, a second intravenous dose of dexmedetomidine (4 µg) was administered. Once the patient was adequately sedated (10 min after starting the remimazolam infusion), the procedure was started. Two minutes later, the remimazolam infusion was reduced to 7 µg/kg/min followed by 3,000 units of heparin, which was administered through the same intravenous site. The remimazolam infusion was reduced again to 5 µg/kg/min 13 min later and then discontinued at the completion of the procedure. Shortly thereafter, the patient began to respond appropriately to verbal and tactile stimuli. When the surgical drapes were removed, a local reaction above the site of the intravenous cannula along the path of the vein was noted, including 0.5-cm non-erythematous, non-pruritic, raised urticarial lesions. As the reaction was noted only above the site of the intravenous cannula and there was no evidence of local mechanical irritation related to the surgical drapes, we assumed that this may have represented an allergic reaction to an intravenous medication. All vitals remained normal and the lungs were clear to auscultation. Over the ensuing 60 min, the urticaria resolved. Considerations as to the etiology of the local urticarial reaction focused on a possible drug allergy. Intraoperatively, the only medications administered included midazolam, dexmedetomidine, remimazolam, and heparin. Although a topical agent (chlorhexidine) was used for skin preparation prior to placement of the intravenous cannula, and a topical cleansing agent and local anesthetic were administered over the femoral vessels prior to vascular access, the urticaria occurred only along the tract of the vein, thereby suggesting that the urticarial reaction was related to an intravenous medication. Given the temporal association, our clinical experience, and review of reports from the literature, we would postulate that the most likely inciting agent was remimazolam. The post-procedure course was unremarkable, and the patient was discharged home. During a subsequent anesthetic for sigmoidoscopy, the patient received midazolam (2 mg) intravenously as part of the anesthetic without issue. Remimazolam was not administered at that time.

## Discussion

Following its introduction for procedural sedation in adults, the clinical use of remimazolam expanded to include primary and adjunctive roles in general anesthesia in adults, and later, a range of applications in pediatric patients [1]. To date, many of the reports of remimazolam use in pediatric-aged patients are anecdotal and involve use in patients with comorbid disease processes commonly affecting the muscle and/or mitochondria (MELAS, mitochondrial myopathies, medium-chain acyl-CoA dehydrogenase deficiency, malignant hyperthermia, muscular dystrophy, stiff person syndrome) or in patients with cardiac conduction pathologies [1]. These anecdotal reports have not identified any clinically significant safety concerns or adverse effects. Given the limited pediatric data on this novel medication, it is important to evaluate all reported adverse effects across populations, recognizing that both the frequency and nature of adverse events in children may differ from those observed in adults [4].

The early literature demonstrated a strong safety profile for remimazolam when compared to other commonly used sedatives including propofol, dexmedetomidine, and midazolam. In a randomized control trial comparing remimazolam with midazolam for procedural sedation for high-risk colonoscopy in adults, the incidence of commonly reported adverse effects including hypotension, hypertension, respiratory depression, and bradycardia was similar or lower to the midazolam group [5]. Similarly, in a randomized phase IIa study analyzing adverse effects of remimazolam compared to midazolam in endoscopy patients, there was a similar rate of adverse events in patients receiving either remimazolam (bolus doses ranging from 0.10 to 0.20 mg/kg) or midazolam (bolus dose of 0.075 mg/kg) [6].

In a meta-analysis comparing remimazolam and propofol for sedation in gynecologic surgery, adverse events including hypotension, bradycardia, injection pain, postoperative nausea, vomiting, dizziness, and respiratory depression were reported. All were less common in the remimazolam group compared to the propofol group [7]. Notably, no anaphylaxis-type reactions were reported in this meta-analysis. Additional information regarding the adverse effect profile of remimazolam was presented in a 2023 scoping review, which included a total of 6,806 patients across 36 case reports and 73 clinical trials [8]. Hypotension was reported in 911 cases, delayed emergence in 68 cases, anaphylaxis in 10 cases, and re-sedation in eight cases. The authors noted that the incidence of hypotension was lower than that observed with other anesthetic agents, even among high-risk patients. Anaphylaxis or severe allergic reactions were identified in only 10 patients, with no reports of isolated cutaneous or other milder allergic manifestations. In the reported anaphylaxis cases, profound hypotension was the predominant clinical feature, with at least two patients requiring cardiopulmonary resuscitation, including chest compressions. Cutaneous signs, such as facial flushing or erythema, were documented in only four patients. Epinephrine was administered in all cases to restore hemodynamic stability, and serum tryptase levels were elevated in eight of the 10 patients. Importantly, the review did not describe patients with less severe allergic presentations, such as isolated cutaneous findings, as observed in our patient.

The initial clinical experience with remimazolam in pediatric-aged patients has demonstrated a similar safety profile. In a comparison of remimazolam to propofol for sedation for magnetic resonance imaging (MRI), the incidence of hypoxemia, hypotension, bradycardia, and injection pain were higher with propofol than with remimazolam [9]. A subsequent randomized controlled trial in children comparing remimazolam with propofol during adenotonsillectomy demonstrated no difference between the two groups in the incidence of bradycardia, tachycardia, hypertension, respiratory depression, excessive sedation, nausea and vomiting, while hypotension and injection site pain were reported significantly less often with remimazolam than propofol [10].

According to FDA prescribing information, the most common adverse effects with Byfavo^®^ (remimazolam) are hypotension, hypertension or hypoxia [11]. Less commonly reported adverse effects include bradycardia, tachycardia, changes in respiratory rate, nausea, pyrexia, and headache. Importantly, there are no reported hypersensitivity reactions in the FDA prescribing information. A detailed analysis performed in 2025 based on the FDA Adverse Event Reporting System (FAERS) outlined safety signals for remimazolam [12]. The FAERS database is a valuable tool for monitoring the safety profiles of newly approved medications, particularly for identifying rare or delayed adverse events that may be missed in clinical trials. It contains millions of voluntarily submitted adverse event reports from clinicians, manufacturers, and patients or caregivers. However, because the reports lack information on concurrent exposures and are subject to reporting bias, FAERS data cannot establish causality or be used to determine incidence. For remimazolam, the FAERS suggested strong signals for clinically significant hypersensitivity reactions including both anaphylactic shock and anaphylactoid reaction. While not implying a causal relationship, it underscores the need for ongoing vigilance with regard to potential allergic reactions when administering remimazolam.

To date, reports in the literature regarding allergic reactions with remimazolam including anaphylaxis or anaphylactoid reactions are rare. Our review of the literature found a total of 15 patients in 10 reports with suspected or confirmed allergic reactions ranging from isolated cutaneous findings to severe hypotension and cardiac arrest (Table 1) [13–22]. The majority of the reactions in these cases involved hemodynamic instability (hypotension); however, some of these events progressed to cardiac arrest. Cutaneous reactions were also identified, including facial or truncal erythema, flushing, rash, and periorbital edema, while the incidence of respiratory effects (hypoxemia, bronchospasm, or airway edema) was low. The tryptase level was elevated in 10 of the 15 cases of suspected allergic reactions, three patients had a positive skin test, and a causal relationship was demonstrated by a positive provocation test in one patient. Despite the severity of the reactions in many cases, all of the patients experienced resolution of their symptoms with interventions, including the early administration of epinephrine, vasoactive agents, antihistamines, intravenous fluids, and respiratory support.

**Table 1:** Previous Reports of Allergic and Anaphylactoid Reactions With Remimazolam

Author and reference	Demographic data and procedure	Reaction noted	Treatment/outcomes
Tsurumi et al, 2021 [13]	32-year-old male undergoing hand surgery	Hypotension, hypoxemia, facial flushing	Ephedrine, epinephrine, endotracheal intubation/returned to baseline status
Uchida et al, 2022 [14]	74-year-old male with severe burn injury undergoing debridement and skin grafting	Hypotension, hypoxemia in patient 1	Norepinephrine, epinephrine, crystalloid/returned to baseline in patient 1.
	59-year-old male undergoing laparoscopic-assisted sigmoid colectomy	Hypotension, tachycardia in patient 2	Ephedrine, phenylephrine, epinephrine/returned to baseline in patient 2.
Yamaoka et al, 2022 [15]	78-year-old male with colon cancer undergoing bowel resection surgery	Hypotension, hypoxemia, bronchospasm	Ephedrine, phenylephrine, norepinephrine, IV fluids/returned to baseline
Hasushita et al, 2022 [16]	72-year-old male undergoing video-assisted thoracoscopic left-lung segmentectomy	Abdominal erythema, hypotension, cardiac arrest	Chest compressions, 100% oxygen ventilation, fluid resuscitation, epinephrine/resuscitation successful and return to baseline
Kim et al, 2022 [17]	Case series of five patients with ages ranging 23–69 years undergoing inguinal herniorrhaphy, umbilical herniorrhaphy, ileostomy takedown, ileocecal resection, thyroidectomy	Cardiovascular collapse, ST-segment elevation, cardiac arrest, tachycardia, dyspnea, cough, chest tightness, facial flushing and skin rash	Vasoactive agents, including ephedrine, phenylephrine, and epinephrine; isotonic fluids; standard ACLS resuscitation/returned to baseline in all five patients.
Hu et al, 2023 [18]	41-year-old male undergoing colonoscopy	Facial erythema, periorbital edema, epiglottic edema, hypotension	Epinephrine/returned to baseline
Lee et al, 2023 [19]	51-year-old male undergoing humeral pinning	Hypotension, rash on chest	Dexamethasone, epinephrine infusion/returned to baseline; remimazolam intravenous provocation test positive
Nakai et al, 2024 [20]	75-year-old male with gastric cancer undergoing robot-assisted gastrectomy	Tracheal and bronchial edema, rash on abdomen and thighs, hypotension, cardiac arrest	Ephedrine, phenylephrine, chest compressions/successful resuscitation, returned to baseline
Mani et al, 2024 [21]	77-year-old male with mastocytosis undergoing central line placement	Hypotension, hyperventilation, tachycardia, hypoxia	Dexamethasone, diphenhydramine, famotidine, phenylephrine, high-flow oxygen/returned to baseline
Rita et al, 2025 [22]	61-year-old female undergoing colonoscopy	Erythema of the trunk	Clorfenamine, hydrocortisone/returned to baseline

IV: intravenous; ACLS: advanced cardiovascular life support.

Another question yet to be answered is the potential for cross reactivity between remimazolam and midazolam. Because of the structural similarity between the two agents, it has been hypothesized that cross-reactivity may exist [23]. Although the incidence of documented allergy to midazolam is exceedingly low, with fewer than 20 reported cases in the literature, prior exposure to midazolam may represent the sensitizing event in the patients with subsequent anaphylaxis to remimazolam. If this cross-reactivity were clinically significant, it might be expected that there be strong, consistent evidence of midazolam reactivity following an allergic response to remimazolam. However, only one of the five patients with reported anaphylactic reactions underwent midazolam skin prick testing (result was positive) following a positive test for remimazolam [8]. However, in that case, the concentration of midazolam used for the skin prick test was high and therefore, the positive reaction was considered to be a false positive related to the irritative nature of the higher concentration.

A key confounding factor is that remimazolam contains dextran-40 as an excipient to enhance solubility. Dextran-40 is a recognized cause of hypersensitivity reactions and may therefore be the offending agent in some cases; because it is not present in midazolam, cross-reactivity between the two drugs would not be expected [21]. Dextran-associated reactions can be clinically indistinguishable from immunoglobulin E (IgE)-mediated anaphylaxis but are often driven by non-IgE mechanisms involving complement activation by immune complexes [20]. Circulating IgG antibodies to dextran are common in the general population, potentially due to exposure to structurally similar food antigens [8, 17]. Patients summarized in Table 1 [13–22] either did not undergo dextran-40 skin testing or had negative results; however, given the frequency of non-IgE-mediated reactions, skin prick testing may lack sensitivity and fail to detect clinically relevant hypersensitivity [17].

### Learning points

In summary, our case potentially represents a local hypersensitivity reaction to remimazolam in an adolescent patient. Although we cannot definitively identify remimazolam as the cause of the hypersensitivity reaction and no formal allergy testing was completed, the temporal association of its administration with the development of the local reaction suggests a possible relationship. During the procedure, only four medications were administered: midazolam, remimazolam, heparin, and dexmedetomidine. Given the temporal association with administration, we identified remimazolam as the most likely etiologic agent responsible for the local allergic reaction. When reviewing the medication list, a local reaction to unfractionated heparin should also be considered [24]. Unlike the more commonly seen delayed-type hypersensitivity reaction (type IV), which occurs days after subcutaneous injection, urticarial reactions typically occur shortly after intravenous administration and generally represent a type I (IgE-mediated) hypersensitivity reaction. Historically, some immediate intravenous reactions were linked to contaminants (chondroitin sulfate) rather than the heparin molecule itself. If such reactions are suspected or confirmed, alternative anticoagulants may be required for future procedures. In clinical situations where additional information is required concerning the etiology, formal allergy consultation and skin testing is recommended.

Remimazolam is a novel sedative agent with an overall favorable safety profile; however, anecdotal reports have described rare but potentially severe hypersensitivity reactions, including both IgE-mediated anaphylaxis and non-IgE-mediated anaphylactoid reactions. Reported clinical manifestations most commonly involve major cardiovascular instability, such as hypotension, shock, or cardiac arrest, with respiratory or cutaneous findings occurring less frequently. Given these concerns, heightened vigilance is warranted when administering this novel pharmacologic agent to ensure comprehensive evaluation of its adverse-effect profile. Prompt recognition of hypersensitivity reactions, immediate discontinuation of remimazolam, and early administration of epinephrine are critical to achieving favorable outcomes. Measurement of serum tryptase levels in the acute setting may aid in confirming an allergic reaction, and delayed skin testing may help identify the offending agent. Given the mild presentation and rapid resolution without progression to systemic signs, a serum tryptase was not measured in our patient and formal allergy testing was not pursued. Although dextran-40, an excipient in the remimazolam formulation, has been proposed as a potential trigger, further investigation is needed to clarify the causative agent and to determine whether cross-reactivity with midazolam exists.

## Data Availability

The data supporting the findings of this case report are available from the authors.
